# Spectroscopic Properties and Biological Activity of Fluphenazine Conjugates with Gold Nanoparticles

**DOI:** 10.3390/molecules29245948

**Published:** 2024-12-17

**Authors:** Oliwia Kowalska, Natalia Piergies, Anna Barbasz, Piotr Niemiec, Patrycja Gnacek, Dorota Duraczyńska, Magdalena Oćwieja

**Affiliations:** 1 Jerzy Haber Institute of Catalysis and Surface Chemistry, Polish Academy of Sciences, Niezapominajek 8, PL-30239 Krakow, Poland; oliwia.kowalska@ikifp.edu.pl (O.K.); patrycja.gnacek@ikifp.edu.pl (P.G.); dorota.duraczynska@ikifp.edu.pl (D.D.); 2 Institute of Nuclear Physics, Polish Academy of Sciences, PL-31342 Krakow, Poland; natalia.piergies@ifj.edu.pl; 3 Department of Biochemistry and Biophysics, Institute of Biology and Earth Sciences, University of the National Education Commission, Podchorazych 2, PL-30084 Krakow, Poland; anna.barbasz@uken.krakow.pl; 4 Faculty of Mathematics and Natural Sciences, Department of Chemistry, University of Applied Sciences in Tarnow, Mickiewicza 8, PL-33100 Tarnow, Poland; p_niemiec@atar.edu.pl

**Keywords:** fluphenazine (FPZ), gold nanoparticles (AuNPs), fluphenazine conjugates, neuroleptics, phenothiazines (PTZ), SH-SY5Y human neuroblastoma cells

## Abstract

Fluphenazine (FPZ) is a well-known neuroleptic that has attracted considerable scientific interest due to its biocidal, virucidal, and antitumor properties. Although methods for encapsulating and delivering FPZ to enhance its activity and reduce side effects have been developed, there is still limited knowledge about its conjugates with gold nanoparticles (AuNPs). Therefore, the aim of this research was to develop a preparation method for stable FPZ-AuNP conjugates and to investigate their physicochemical and biological properties. FPZ-AuNP conjugates were synthesized via a ligand exchange process on the surface of gold nanoparticles (AuNPs) with an average size of 17 ± 5 nm. Electrokinetic measurements revealed that the zeta potential of FPZ-AuNPs is affected by both their composition and pH. The FPZ-AuNPs exhibited an isoelectric point due to the acid–base properties of FPZ. Surface-enhanced Raman spectroscopy (SERS), combined with density functional theory (DFT), was used to determine the adsorption structure of FPZ after conjugation. Studies with human neuroblastoma cells (SH-SY5Y) revealed that FPZ-AuNP conjugates more effectively reduced cell viability compared to citrate-stabilized AuNPs alone or free FPZ molecules. The reduction in SH-SY5Y cell viability was found to be dependent on the FPZ-AuNP concentration.

## 1. Introduction

**Figure 1:**
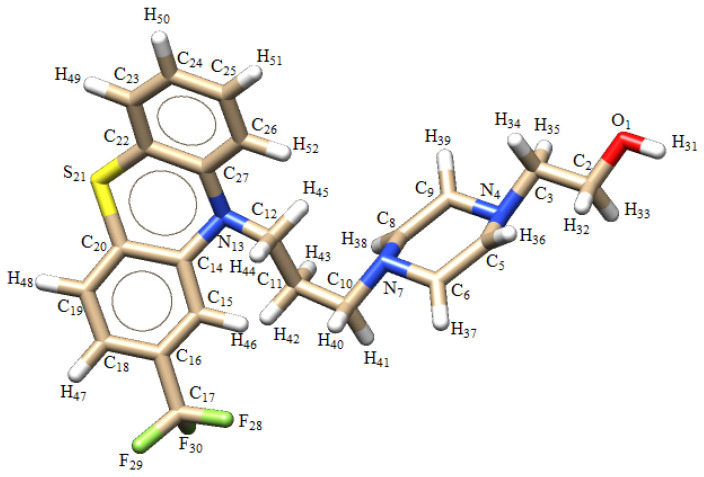
Chemical structure of fluphenazine (FPZ).

Fluphenazine (FPZ) is an antipsychotic drug widely used in the treatment of various mental disorders, such as schizophrenia [1]. It has been shown to effectively reduce hallucinations, delusions, or episodes of strange behavior in patients with schizophrenia and other mental disorders [1]. The biological properties and medical application of FPZ are similar to those of chlorpromazine (CPZ). Both FPZ and CPZ are first-generation neuroleptics and are derivatives of phenothiazine (PTZ). From a chemical perspective, the name of FPZ is 2-[4-[3-[2-(trifluoromethyl)-10*H*-phenothiazin-10-yl]propyl]piperazin-1-yl]ethanol, which indicates that it possesses a piperazine side chain attached to the nitrogen in the para-thiazine ring and a trifluoromethyl group attached to the second benzene ring (Figure 1), which increases the efficacy of the drug [1,2].

FPZ is a drug commonly administered either orally, typically as the dihydrochloride salt, or intramuscularly as a long-acting decanoate or enanthate ester in oil. It is a first-generation antipsychotic that functions by blocking postsynaptic dopamine D2 and D1a receptors [3,4]. Its use is linked to various side effects, including orthostatic hypotension and reflex tachycardia due to alpha-adrenergic blockade, as well as anticholinergic and extrapyramidal symptoms such as tardive dyskinesia, muscle rigidity, tremors, dystonia, and akathisia [5]. Additionally, it can cause neuroleptic malignant syndrome [6,7]. Pharmacokinetically, oral FPZ exhibits considerable inter-individual variability because it undergoes “first-pass” metabolism in the liver before entering the systemic circulation. Its serum half-life ranges from 15 to 30 h, with the peak plasma concentration being reached within a few hours of administration [8].

Although the pharmacokinetic properties, mechanism of action, and side effects of FPZ have been extensively described in numerous literature reports, the scientific interest in this neuroleptic remains high. Ongoing research seeks to further explore its biological activity and uncover potential applications that are not yet fully realized. In the past year, scientists have focused on the bactericidal [9,10,11,12,13], virucidal [14,15,16], and antitumor [17,18,19], properties of FPZ. Furthermore, some literature reports suggest that PTZ could be promising candidates in the treatment of neurodegenerative diseases such as Parkinson’s and Alzheimer’s [13,20].

Generally, it has been found that several phenothiazine-derived drugs, including FPZ, can potentiate the activities of antibiotics used to treat infections caused by both Gram-positive and Gram-negative bacteria [12]. Moreover, some evidence suggests that phenothiazines possess inherent antibacterial and efflux inhibitory properties, which may enable them to combat drug resistance [12].

Several studies indicate that FPZ shows promise as a potential cancer therapy as it has been found to reduce the viability of various cancer cell lines [1,2,17,18,19]. Its cytotoxic effects have been observed in cancers such as lung, breast, colon, liver, brain, oral, ovarian, and skin cancers, as well as leukemia [1,2]. FPZ appears to influence the cell cycle, inhibit cell proliferation, and induce apoptosis in multiple cancer types. Additionally, it has been shown to target cancer-related proteins like ABCB1 and P-glycoprotein while also modulating the Akt and Wnt signaling pathways. Some research also suggests that FPZ induces DNA changes, disrupts cell invasion and migration, and affects ROS production [1,2,21,22]. These findings highlight FPZ as a potentially valuable compound for further cancer therapy research.

Due to the wide range of biological activities and excellent therapeutic potential of FPZ, numerous methods of drug administration have been developed. For instance, Dunne et al. [23] proposed the encapsulation of FPZ into biodegradable poly-lactide-co-glycolide (PLGA)-based microparticles. Their research demonstrated that the release of the drug from capsules could be controlled through a combination of FPZ diffusion and degradation-assisted breakdown of the polymer forming the capsules. Similarly, Ershaid et al. focused on developing transdermal FPZ formulations [24]. They proposed encapsulating FPZ in three types of microneedle (MN) systems using PLGA-tipped MNs. These microsystems were then applied to create effective microneedle array patches for the sustained delivery of FPZ.

An analysis of the literature revealed that while significant attention has been devoted to encapsulating FPZ into degradable microcapsules, comparatively less focus has been placed on conjugating FPZ to the surfaces of hard micro- and nanoparticles. This topic is particularly intriguing in the context of FPZ conjugation with plasmonic nanoparticles, such as silver, gold, and platinum. To the best of our knowledge, no scientific articles have been published on the preparation of stable FPZ conjugates with gold nanoparticles (AuNPs). Developing a method for preparing stable FPZ-AuNP conjugates could open new avenues for drug delivery and provide insights into the drug’s behavior and transformations at the cellular level. AuNPs are promising candidates for such conjugation due to their low toxicity, which is surface- and size-dependent, as well as their plasmonic properties, which are utilized in advanced spectroscopic techniques like surface-enhanced Raman spectroscopy (SERS) and surface-enhanced infrared absorption spectroscopy (SEIRA). The conjugation of FPZ with AuNPs could result in nanostructures with biological activity arising from the combined properties of both components. Additionally, techniques such as SERS and SEIRA can be employed to determine the adsorption pattern of FPZ on the AuNP surface and to monitor drug release over time.

Considering the issues described above, the aim of this study was to develop a preparation procedure for stable FPZ-AuNP conjugates and conduct detailed electrokinetic and spectroscopic analyses of these nanostructures. Particular attention was paid to determining the impact of the FPZ concentration and solution pH on the electrokinetic properties of the FPZ-AuNP conjugates. Moreover, it was hypothesized that a spectroscopic evaluation of the adsorption pattern of FPZ immobilized on AuNP surfaces could provide insights into potential changes in the biological activity of FPZ after conjugation. To assess the biological activity of the formed conjugates, this study focused on evaluating changes in the viability of SH-SY5Y human neuroblastoma cells following FPZ-AuNP treatment.

The main research hypothesis proposed that the electrokinetic properties and toxicity of FPZ-AuNP conjugates would depend on their composition, with the FPZ content being a key influencing factor. This hypothesis was tested through measurements of the electrophoretic mobility of FPZ-AuNP conjugates and an analysis of Raman (RS) and surface-enhanced Raman spectroscopy (SERS) spectra. Furthermore, changes in mitochondrial activity in SH-SY5Y cells, evaluated using an MTT assay, were used to verify the assumption regarding the biological activity of the FPZ-AuNP conjugates.

## 2. Results and Discussion

### 2.1. Physicochemical Characteristics of TC-AuNPs and FPZ-AuNP Conjugates

**Figure 2:**
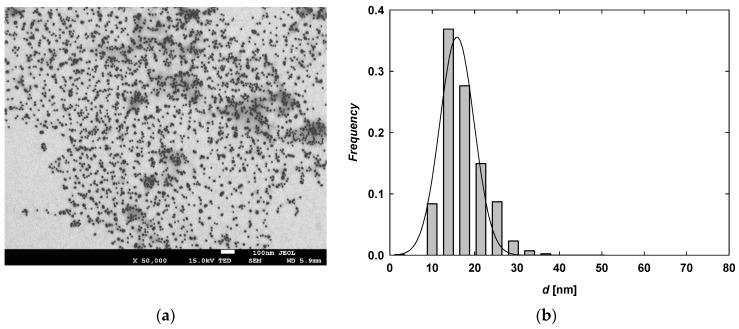
(**a**) Typical TEM micrograph showing TC-AuNPs and (**b**) size distribution of TC-AuNPs generated from analysis of several TEM micrographs.

The pH and conductivity of purified citrate-stabilized AuNP (TC-AuNP) suspensions were 5.8 and 30 µS/cm, respectively. The mass concentration of gold in the purified suspension was 185 mg/L. TC-AuNPs were quasi-spherical and monodisperse, as confirmed by the recorded TEM micrographs. The average size of the TC-AuNPs was determined to be 17 ± 5 nm with a polydispersity index (PdI) of 0.29. A typical TEM micrograph and the size of TC-AuNPs are presented in Figure 2. The maximum absorption band of the TC-AuNP suspension, arising from localized surface plasmon resonance (LSPR), was observed at a wavelength of 525 nm. The position of this band was independent of the pH of the suspension (Figure S1, Supplementary Materials). At pH 5.8 and a temperature of 25 °C, the TC-AuNPs remained stable across a broad range of ionic strengths. Aggregation, indicated by an increase in the hydrodynamic diameter, was observed at an ionic strength of 5 × 10^−2^ M (Figure S2a, Supplementary Materials). These results are consistent with other literature reports on citrate-stabilized AuNPs [25,26,27]. Measurements of the hydrodynamic diameters of TC-AuNPs, conducted at ionic strengths of 10^−3^ M and 10^−2^ M across a pH range of 2–11, further confirmed their stability (Figure S2a, Supplementary Materials).

It is well known that citrate-stabilized AuNPs carry a negative charge [26,27,28,29]. It was observed that the zeta potential of TC-AuNPs decreases significantly with an increasing pH (Figure S3, Supplementary Materials). For instance, at an ionic strength of 10^−2^ M, the zeta potential of TC-AuNPs was measured as −33 ± 3 mV at pH 3 and −63 ± 2 mV at pH 11.

Although citrate-stabilized AuNPs are well known and widely described in numerous literature reports, this physicochemical characteristic analysis was conducted to precisely determine the range of their stability and to select the optimal condition for the ligand exchange process and the formation of FPZ conjugates. The chemical compound FPZ is usually available in the form of its dichloride salt [C_22_H_28_Cl_2_F_3_N_3_OS]. As a result, aqueous solutions of FPZ salt are electrolytes with concentration-dependent conductivity and ionic strength. Given this fact, as well as the ionic strength-dependent stability of TC-AuNPs (Figure S2, Supplementary Materials), one can predict the maximum concentration of FPZ for the conjugation process, which should not induce the aggregation process of TC-AuNPs. Considering the optimal FPZ concentration range for the ligand exchange process, it is important to note that FPZ is an amphiphilic compound and exhibits a high propensity toward aggregation, especially at high concentrations [30]. It has been reported that the critical micelle concentration of FPZ is 11 mM [31]. On the other hand, the acid–base properties of FPZ are crucial for effective adsorption onto the surface of TC-AuNPs and the formation of stable FPZ-AuNP conjugates. It is well known that FPZ has three values of pKa: 3.65, 5.95, and 7.93 [32]. The pKa values are associated with protonation–deprotonation at the nitrogen atom of the phenothiazine ring and the protonation and deprotonation of the piperidine moiety [32]. For instance, at a physiological pH, the distal nitrogen will be uncharged, while the proximal nitrogen will be approximately 90% protonated [33]. Taking this into account, to enhance the electrostatically driven adsorption of FPZ on the surface of TC-AuNPs, mild acidic conditions should be maintained.

Aqueous solutions of FPZ exhibit characteristics bands in the UV-vis region. The concentration-dependent absorption spectra of FPZ solutions revealed one band at a wavelength of 256 nm and another at 306 nm (Figure S4, Supplementary Materials). Moreover, it was established that the position and intensity of these bands are only weakly influenced by the pH of the solution (Figure S5, Supplementary Materials).

As previously mentioned, the FPZ-AuNP conjugates were prepared in acidic conditions by mixing an appropriate volume of purified TC-AuNP suspension with a specified volume of FPZ solutions at a controlled molar concentration. The preparation conditions were optimized to achieve FPZ-AuNP conjugates with a constant TC-AuNP mass concentration of 50 mg/L and varying FPZ concentrations. The formation of FPZ-AuNP was monitored using spectroscopic and electrokinetic measurements. The absorption spectra recorded for FPZ-AuNP conjugates with controlled compositions are shown in Figure S6 (Supplementary Materials). Analyzing the recorded spectra revealed that the intensity of two bands originating from FPZ molecules increased significantly with a rising FPZ concentration. However, the positions of these bands remained unchanged. A slightly different trend was observed for the characteristic band of TC-AuNPs. Both the position and intensity of this band were strongly influenced by the FPZ concentration. For FPZ concentrations ranging from 10^−6^ to 5 × 10^−5^ M, the intensity of the band at 525 nm decreased, and an additional band appeared at the 652–702 nm region. In contrast, for FPZ concentrations between 10^−4^ M and 5 × 10^−3^ M, the band underwent a slight bathochromic shift to 528 nm.

The spectroscopic features of FPZ-AuNP conjugates observed in the UV-vis region closely resemble those reported for CPZ-AuNP conjugates in a previous study [27]. Consequently, it can be concluded that stable FPZ-AuNP conjugates are formed only at a specific concentration ratio of FPZ to TC-AuNPs.

**Figure 3:**
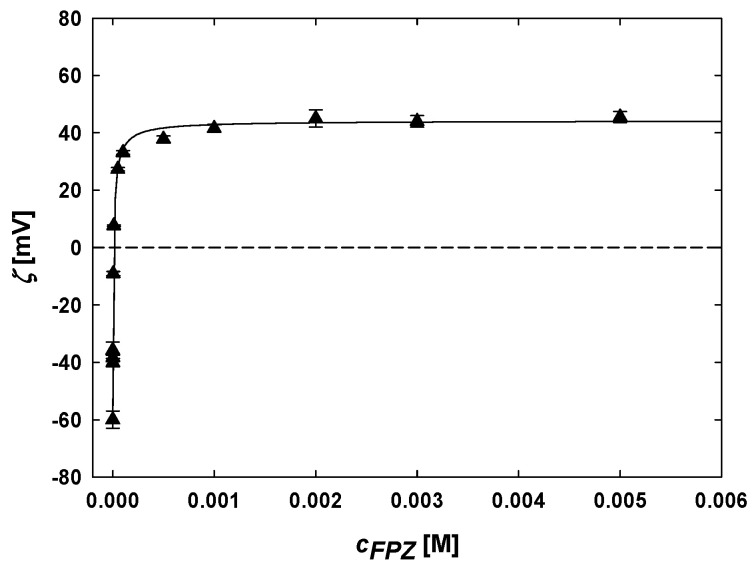
The dependence of the zeta potential of TC-AuNPs on the FPZ concentration in the suspension. The deposition conditions are as follows: TC-AuNP concentrations of 50 mg/L (▲), a pH range of 5.7–3.2, and a temperature of 25 °C. The solid line serves as a guide for the eyes.

Electrokinetic studies on the formation of FPZ-AuNP conjugates were conducted by measuring their electrophoretic mobility and subsequently calculating the zeta potential using Henry’s model. The results, presented in Figure 3, illustrate the relationship between the zeta potential of TC-AuNPs and the FPZ molar concentration. Analyzing the data revealed that increasing the FPZ concentration leads to a progressive rise in the zeta potential of TC-AuNPs. Initially, TC-AuNPs exhibited a zeta potential of −60 ± 3 mV. The addition of FPZ and the subsequent formation of FPZ-AuNP conjugates at an FPZ concentration of 10^−^⁷ M resulted in an increase in the zeta potential to −36 ± 3 mV. This change was attributed to the adsorption of positively charged FPZ cations, reducing the negative charge on the TC-AuNPs. The inversion of the zeta potential occurred at 5 × 10^−6^ M FPZ. Further increases in the FPZ concentration, from 10^−5^ M to 5 × 10^−3^ M, caused the zeta potential to rise from 7 ± 1 mV to 45 ± 2 mV. Additionally, it was observed that increasing the FPZ concentration beyond 10^−3^ M did not result in a further increase in the zeta potential, which stabilized at a constant value for the FPZ-AuNP conjugates.

The results confirm the successful formation of FPZ-AuNP conjugates through the ligand exchange process, as well as the selective adsorption of FPZ cations onto the AuNPs’ surfaces. Moreover, these findings highlight that the electrokinetic properties of FPZ-AuNP conjugates are highly dependent on their composition. Notably, similar observations were reported for CPZ-AuNP conjugates with a controlled composition [27]. FPZ-AuNP conjugates with a low zeta potential (|ζ| < 20 mV) were found to be unstable and prone to aggregation, as confirmed by the absorption spectra recorded in the UV-vis region (Figure S6, Supplementary Materials). However, this study demonstrated that it is possible to prepare both positively and negatively charged FPZ-AuNP conjugates with a desired composition.

Based on the acid–base behavior of FPZ [32,33], it was hypothesized that these properties would influence the formed FPZ-AuNP conjugates. Since FPZ molecules are adsorbed onto the AuNP surface, it can be assumed that changes in the pH of the suspensions would directly affect the electrokinetic properties of the FPZ-AuNP conjugates. To test this hypothesis, three types of conjugates with varying compositions and surface charges were prepared. In these conjugates, the concentration of AuNPs was maintained at 50 mg/L, while the FPZ concentrations were set at 10^−6^ M, 5 × 10^−5^ M, and 10^−4^ M. The zeta potential of the resulting FPZ-AuNP conjugates was then measured under controlled pH conditions, adjusted using hydrochloric acid and sodium hydroxide.

**Figure 4:**
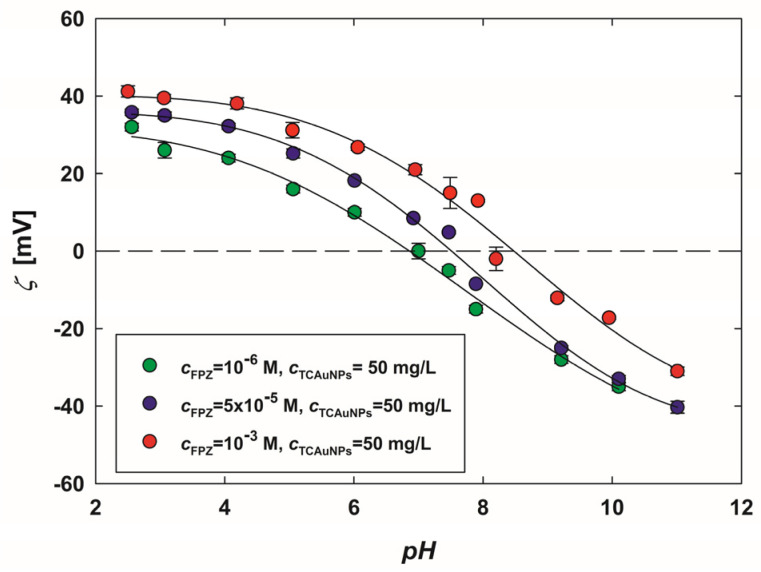
The dependence of the zeta potential of FPZ-AuNP conjugates on the pH, regulated by the addition of HCl or NaOH. The measurements were conducted for FPZ-AuNPs with diverse compositions, with a TC-AuNP concentration of 50 mg/L and FPZ concentrations of (•) 10^−6^ M, (•) 5 × 10^−5^ M, and (•) 10^−3^ M. The solid lines serve as guides for the eyes.

The obtained dependencies are shown in Figure 4. As shown, the zeta potential of each type of FPZ-AuNP conjugate decreases with an increasing pH, displaying similar trends across all samples. For instance, in the conjugates with the highest FPZ concentration, the zeta potential measures 41 ± 2 mV at pH 2.5 and −31 ± 1 mV at pH 11.

Regardless of the composition of the FPZ-AuNP conjugates, their isoelectric point lies within the pH range of 7.5–8.5 (Figure 4), corresponding to the deprotonation of the second nitrogen atom of the piperidine moiety [32,33]. This indicates that the observed decrease in the zeta potential of FPZ-AuNP conjugates is strongly associated with the deprotonation of FPZ molecules. Consequently, it was demonstrated that the electrokinetic properties of FPZ-AuNP conjugates can be effectively modeled based on their composition and pH, aligning with findings previously reported for CPZ-AuNP conjugates [27].

### 2.2. Spectroscopic Characteristics of FPZ and FPZ-AuNP Conjugates

**Figure 5:**
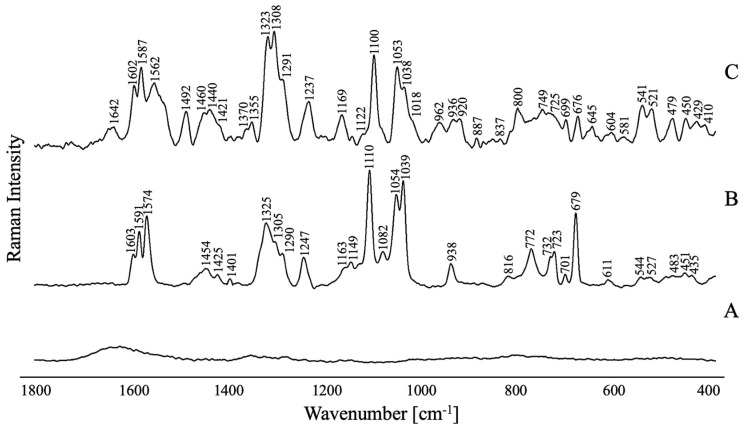
(**A**) The Raman spectrum of the TC-AuNPs in the solution; (**B**) the Raman spectrum of the non-adsorbed FPZ in the solution; and (**C**) the SERS spectrum for FPZ (5 × 10^−5^ M) immobilized on TC-AuNPs (50 mg/L) in the spectral range of 1800–400 cm^−1^.

Figure 5 illustrates the RS spectrum for TC-AuNPs, comparing it with the RS and SERS spectra for FPZ before and after its adsorption on the TC-AuNPs’ surfaces in the spectral range of 1800 cm^−1^ to 400 cm^−1^. The wavenumbers of the observed bands, along with the proposed assignments [27,34,35,36,37,38,39,40,41,42,43,44,45,46,47,48] and full width at half maximum (FWHM), are detailed in Table 1.

As expected, the RS spectra are dominated by the bands corresponding to the modes of the phenothiazine ring (PTZ) moiety (Figure 5). The most intense bands are attributed to the stretching and deformation vibrations of this aromatic moiety and appear at 1603 cm^−1^ [ν_s_(PTZ)], 1591 cm^−1^ [ν_s_(PTZ)], 1580–1574 cm^−1^ [ν(CC)_PTZ_], 1389–1384 cm^−1^ [ν(PTZ)/ρ_b_(CH)_PTZ_], 1325 cm^−1^[ν(CCC)_PTZ_], 1247–1242 cm^−1^ [ρ_b_(CH)_PTZ_], 1149–1141 cm^−1^ [ρ_b_(CH)PTZ], 1111–1110 cm^−1^ [ν_s_(PTZ)], 1039–1038 cm^−1^ [δ_i.p._(CH)_PTZ_], 976 cm^−1^ [δ_oop_(CH)_PTZ_], 772 cm^−1^ [δ_oop_(CH)_PTZ_], 736–723 cm^−1^ [δ_oop_(CH)_PTZ_], 701–693 cm^−1^ [δ_i.p._(CCC)_PTZ_/δ_i.p._(CH)_PTZ_], 679–671 cm^−1^ [ν_s_(PTZ)/ν(CS)/ρ_b_(CH)_PTZ_], 621–611 cm^−1^ [δ(CCC)_PTZ_/δ(CNC)_PTZ_], 550–544 cm^−1^ [ν(SC)_PTZ_/δ_oop_(PTZ)], 483–481 cm^−1^ [ρ_b_(CSC)/δ_oop_(PTZ)], and 435–424 cm^−1^ [δ_oop_(PTZ)].

The spectra also include bands originating from the vibrations of the CF_3_ group. These bands are observed at the ranges of 1082–1080 cm^−1^ [ν_s_(CF_3_)] and 746–732 cm^−1^ [ν_s_(CF_2_)]. Additionally, vibrations of this functional group contribute to the band at the ranges of 1149–1141 cm^−1^ [ν_as_(CF_2_)] and 550–544 cm^−1^ [δ(CF_3_)].

Bands resulting from vibrations of aliphatic groups are also noticeable in the spectra. The bands attributed to the propylene and ethylene bridges are observed in the RS spectra at 1454–1451 cm^−1^ [ρ_s_(CH_2_)], 1401–1392 cm^−1^ [ρ_w_(CH_2_)], 1346–1341 cm^−1^ [ρ_s_(CH_2_)], 1316–1305 cm^−1^ [ρ_t_(CH_2_)], 1300–1290 cm^−1^ [ρ_t_(CH_2_)], 1278–1277 cm^−1^ [δ(CH)_CH2_/ρ_w_(CH_2_)], 1163 cm^−1^ [ρ_w_(CH_2_)], 1135–1131 cm^−1^ [δ(CH)_CH2_/ρ_t_(CH_2_)], and 819–816 cm^−1^ [ρ_r_(CH_2_)/ρ_w_(CH_2_)]. Moreover, the vibrations of the propylene bridge contribute to the bands at 1247–1242 cm^−1^ [ρ_t_(CH_2_)], 1111–1110 cm^−1^ [ρ_t_(CH_2_)], 1055–1054 cm^−1^ [ν(CC)], 772 cm^−1^ [ρ_r_(CH_2_)], and 621–611 cm^−1^ ρ_r_(CH_2_). For the OH group, the bands characteristic for this moiety are visible at 1111–1110 cm^−1^ [ρ_b_(OH)] and 1055–1054 cm^−1^ [ν(OC)]. In the case of the piperazine moiety, the bands associated with it appear at 819–816 cm^−1^, 772 cm^−1^, and 736–723 cm^−1^ and are assigned to the δ_i.p._(PZ) modes, while those observed at 664–663 cm^−1^, 483–481 cm^−1^ [δ_oop_(PZ)], and 456–451 cm^−1^ are attributed to the δ_oop_(PZ). Furthermore, vibrations of this functional group also contribute to the spectral features at 1278–1277 cm^−1^ [ν(CN)_PZ_], 1111–1110 cm^−1^ [ν(CN)_PZ_], 1055–1054 cm^−1^ [ρ_b_(CH)_PZ_], 679–671 cm^−1^ [δ_oop_(PZ)], 550–544 cm^−1^ [δ_oop_(PZ)], 527–525 cm^−1^ [δ_oop_(PZ)], and 435–424 cm^−1^ [δ_oop_(PZ)].

A thorough comparison was conducted between the bands present in the RS and SERS spectra, focusing on their intensity, position, and width. This analysis facilitated the determination of the spatial conformation of the FPZ molecule absorbed on the metallic surface. To ensure a nuanced interpretation of the gathered spectroscopic data, surface selection rules were applied. These rules serve as a guiding framework, elucidating potential alterations and distinctive characteristics within the spectral pattern induced by the molecule’s interaction with the metal substrate. As eloquently detailed by Moskovits et al. [49], vibrations characterized by polarizability derivative components oriented perpendicular to the metal surface notably exhibit the most profound enhancement within the SERS spectrum, providing key insights into the intricate dynamics of molecular interactions at the metal interface.

In the case of the studied FPZ molecule, the most pronounced SERS spectral features correspond to the phenothiazine (PTZ) vibrations. These bands appear at 1602 cm^−1^, 1587 cm^−1^, 1562 cm^−1^, and 1038 cm^−1^ and are assigned to ν_s_(PTZ), ν(CC)_PTZ_, and δ_i.p._(CH)_PTZ_, respectively. Additionally, the observed shifts in position and the broadening of these bands (∆ν ≈ 4 cm^−1^ and ∆FWHM ≈ 8 cm^−1^) indicate a strong interaction between the PTZ group and the surfaces of TC-AuNPs. Data in the literature confirm that PTZ derivatives can interact with AuNPs through the π orbitals of aromatic rings or the lone pair of sulfur, nitrogen, or substituent electrons [50]. The spectral pattern attributed to the CF_3_ moiety suggests that it directly participates in the molecule/metal interaction. Specifically, the bands at 749 cm^−1^, 633 cm^−1^, and 541 cm^−1^ show significant enhancement compared to the corresponding RS spectrum, providing evidence that CF_3_ interacts with AuNPs and induces a perpendicular orientation of the PTZ on the metal nanosurface.

The interaction between the CH_2_ groups of FPZ and the AuNP surface is also evident. In the SERS spectrum, the vibration bands corresponding to the propylene and ethylene bridges are visible at 1460 cm^−1^, 1308 cm^−1^, 1291 cm^−1^, 1169 cm^−1^, and 1053 cm^−1^ and are assigned to the ρ_s_(CH_2_), ρ_t_(CH_2_), ρ_t_(CH_2_), ρ_w_(CH_2_), and ν(CC) modes, respectively. A clear enhancement in these bands in the SERS spectrum, compared to their counterparts in the RS spectrum, is accompanied by a shift in position (ν ≈ 6 cm^−1^) and broadening (∆FWHM ≈ 6 cm^−1^). These changes confirm that the CH_2_ groups interact with the AuNPs. Moreover, since the modes characteristic of the OH group also contribute to the bands at 1100 cm^−1^ and 1053 cm^−1^, this functional group may also be involved in the drug/metal interaction.

Additionally, the bands at 656 cm^−1^, 479 cm^−1^, and 450 cm^−1^, assigned to [δ_oop_(Pz)], show stronger intensity compared to the corresponding features in the RS spectrum. This indicates that the piperazine moiety is positioned close to the gold surface, adopting a parallel orientation.

**Figure 6:**
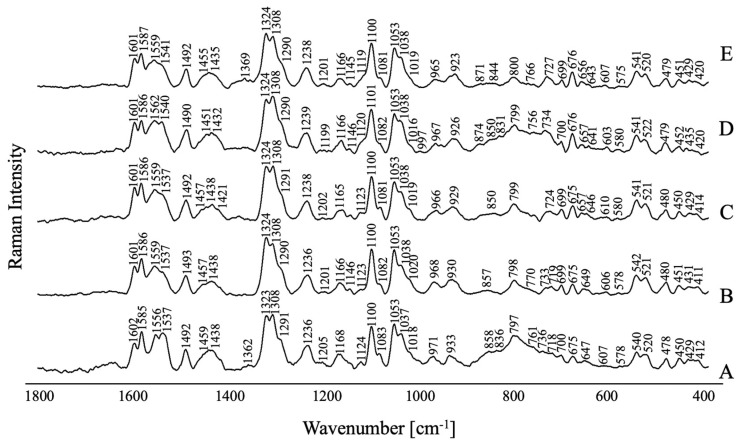
The SERS spectra for FPZ immobilized on the AuNPs measured at pH values of (**A**) 3.0, (**B**) 4.5, (**C**) 5.5, (**D**) 7.5, and (**E**) 9.5 in the spectral range of 1800–400 cm^−1^. The FPZ concentration in the SERS measurements was equal to 5 × 10 ^−4^ M; the concentration of TC-AuNPs was equal to 50 mg/L.

Figure 6 illustrates the SERS spectra of FPZ adsorbed on the AuNP surface under different pH conditions. No significant changes are observed in the recorded spectra, which indicates that the pH value has not affected the stability of FPZ-AuNP conjugates or the way these systems interact with the metal surface.

**Figure 7:**
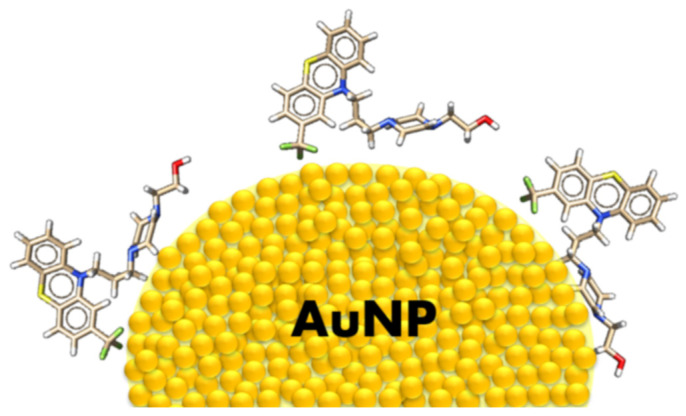
The suggested orientation of FPZ on the surface of AuNPs. A single yellow ball represents an individual gold atom.

The adsorption geometry of the FPZ molecule on the AuNP surface is illustrated in Figure 7.

### 2.3. Biological Activity of FPZ, TC-AuNPs, and FPZ-AuNP Conjugates Towards SH-SY5Y Cell Line

The evaluation of the toxicity of free and conjugated FPZ towards human neuroblastoma cell lines (SH-SY5Y) was the final step of the studies. It is worth noting that the impact of FPZ on SH-SY5Y cells has been extensively documented in the literature [2,18,51,52,53,54]. Thus, this cell line was chosen for studying the cytotoxicity of FPZ conjugated with AuNPs.

**Figure 8:**
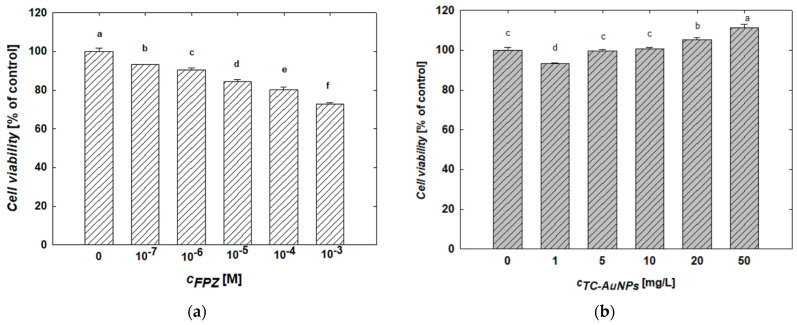
The effect of (**a**) FPZ and (**b**) TC-AuNPs on the viability of SH-SY5Y cells. The viability of the cells was evaluated after 24 h of exposure to FPZ and TC-AuNPs using an MTT assay. The letters indicate significant (*p* < 0.05) differences between the results of each treatment.

The MTT assay was used to evaluate changes in the viability of SH-SY5Y cells after 24 h of treatment with FPZ solutions at controlled concentrations. The results of this study are presented in Figure 8a. An analysis of the data reveals a monotonic decrease in SH-SY5Y cell viability with an increasing FPZ concentration. For example, treatment with 10^−6^ M and 10^−3^ M FPZ solutions reduced cell viability to 94% and 73%, respectively. These results are consistent with the data reported by Gil-Ad et al. [52], who also observed a concentration-dependent decrease in SH-SY5Y viability and determined an IC_50_ value of 15 ± 1.6 µM.

The effect of TC-AuNPs on the viability of SH-SY5Y cells after 24 h of treatment was also evaluated using the MTT assay. The results are presented in Figure 8b. It was found that increasing the TC-AuNP concentration promotes cell proliferation. After 24 h of exposure, the viability of cells treated with TC-AuNPs at concentrations of 1 mg/L and 50 mg/L were 93% and 111%, respectively. It is worth mentioning that the increase in SH-SY5Y cell viability as a result of the AuNP treatment has also been reported by other researchers [55,56,57]. However, the observed effect is highly dependent on the size and surface properties of AuNPs, as discussed in detail in numerous literature reports [56,57].

In the next phase of the investigation, three types of FPZ-AuNP conjugates with controlled compositions and electrokinetic properties were prepared to evaluate their cytotoxicity. These conjugates, designated as FPZ-AuNP1, FPZ-AuNP2, and FPZ-AuNP3, were formulated with adjusted concentrations of AuNPs and varying amounts of FPZ. The concentrations of both components were selected so that, in cell experiments, the highest AuNP concentration in the conjugates was 50 mg/L, while the FPZ concentrations were 10^−6^ M, 5 × 10^−5^ M, and 10^−3^ M for FPZ-AuNP1, FPZ-AuNP2, and FPZ-AuNP3, respectively.

**Figure 9:**
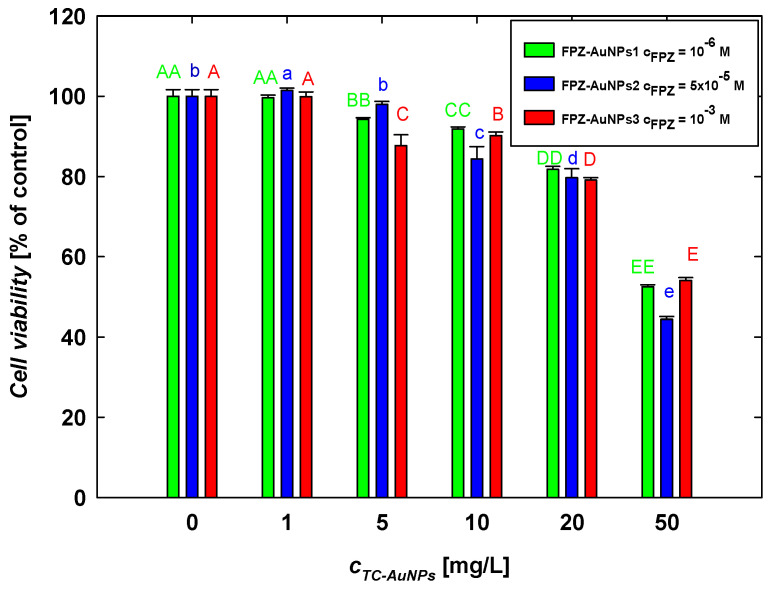
The effect of FPZ-AuNP conjugates with diverse compositions on the viability of SH-SY5Y cells. The viability of the cells was evaluated after 24 h of exposure to FPZ-AuNP conjugates using an MTT assay. The letters indicate significant (*p* < 0.05) differences between the results of each treatment.

Due to their differing compositions, the FPZ-AuNP1, FPZ-AuNP2, and FPZ-AuNP3 conjugates exhibited zeta potentials of −10 ± 2 mV, 5 ± 1 mV, and 15 ± 4 mV, respectively, at pH 7.4 (Figure 4). Each type of conjugate was diluted to assess its concentration-dependent cytotoxicity. The results, presented in Figure 9, were analyzed while considering that dilution affected the concentrations of both FPZ and AuNPs in the conjugates.

In general, it was observed that the viability of SH-SY5Y cells decreased with increasing FPZ-AuNP concentrations regardless of their composition. For instance, in FPZ-AuNP conjugates containing 10 mg/L of AuNPs, cell viability ranged from 84% for FPZ-AuNP2 to 91% for FPZ-AuNP1. The most significant decrease in SH-SY5Y cell viability occurred at the highest tested conjugate concentration, specifically those containing 50 mg/L of AuNPs. In this case, the cell viability for FPZ-AuNP1 and FPZ-AuNP2 ranged between 52% and 54%. However, for FPZ-AuNP2, viability further dropped to 44%.

Based on these results, several interesting trends can be observed. First, the conjugation of FPZ with TC-AuNPs significantly increases the toxicity of TC-AuNPs. Although the conjugates contained varying amounts of FPZ and exhibited different surface charges, the effects induced by each type of composite at a given AuNP concentration were comparable. Only minor differences were observed for FPZ-AuNP2 conjugates.

When comparing the results of the SH-SY5Y cell treatment with FPZ (Figure 8) and FPZ-AuNPs (Figure 9), it can be observed that conjugation enhanced the toxic effect of the investigated phenothiazine. For instance, free FPZ at a concentration of 10^−3^ M reduced cell viability to 73%, whereas after conjugation with FPZ-AuNP3, the viability of SH-SY5Y cells decreased to 54%. This effect was less pronounced at lower FPZ concentrations. For example, at a concentration of 10^−4^ M (Figure 8), free FPZ and FPZ-AuNP3 containing 5 mg/L of AuNPs resulted in cell viabilities of 81% and 85%, respectively.

These investigations revealed that the surface charge of FPZ-AuNPs has a relatively minor effect on the viability of SH-SY5Y cells, as determined by the MTT assay. However, considering that FPZ-AuNPs are AuNPs stabilized by low-molecular-weight cationic molecules, their effects can be compared to those induced by negatively charged TC-AuNPs, which are stabilized by citrate anions. TC-AuNPs exhibit a highly negative zeta potential (Figure S3) and are significantly less toxic (Figure 8b). Therefore, it can be concluded that AuNPs with a positive zeta potential are more toxic to SH-SY5Y cells. This finding is consistent with the observations described by Valdiglesias et al. [56].

Nevertheless, the source of this charge—the chemical structure of the molecules stabilizing the AuNPs—appears to play a pivotal role in inducing the biological activity of colloidal systems. This relationship was also observed for both positively and negatively charged AuNPs as well as CPZ conjugates [58].

## 3. Materials and Methods

### 3.1. Reagents

Hydrogen tetrachloroaurate (III) hydrate, trisodium citrate (TC) dihydrate, and sodium chloride were acquired from Sigma Aldrich (Darmstadt, Germany). Sodium hydroxide and hydrochloric acid were supplied by Avantor Performance Materials (Gliwice, Poland). Fluphenazine dihydrochloride (FPZ) was delivered by Pol-Aura Odczynniki Chemiczne (Morąg, Poland). These chemicals were used without further purification. Ultrapure water was obtained with the use of the Milli-Q Elix&Simplicity 185 purification system (Millipore SA, Molsheim, France).

### 3.2. Cell Lines and Cell Culture Reagents

SH-SY5Y human neuroblastoma cell line was obtained from American Type Culture Collection (ATCC) (Manassas, VA, USA). The cells were cultured in DMEM-supplemented medium (with 10% fetal bovine serum (FBS) and 0.01% penicillin-streptomycin) at 37 °C in a humidified atmosphere of 5% CO_2_. The culture media, DMEM, and antibiotics were purchased from PAN-Biotech GmbH (Aidenbach, Germany).

### 3.3. Synthesis of Gold Nanoparticles (TC-AuNPs) and Fluphenazine Conjugates with Gold Nanoparticles (FPZ-AuNPs)

Citrate-stabilized gold nanoparticles (TC-AuNPs) were prepared using a modified version of the Turkevich method [25]. Briefly, 200 mL of a 1 mM aqueous solution of hydrogen tetrachloroaurate was heated to 88 °C. Then, 20 mL of a 39 mM trisodium citrate solution was added to the gold precursor solution while stirring. Heating was maintained for 30 min, although the light yellow solution turned burgundy red 10 min after the addition of trisodium citrate. The resulting TC-AuNP hydrosol was then cooled to room temperature and purified to remove excess ions using an Amicon 8400 ultrafiltration chamber equipped with a regenerated cellulose membrane (Millipore, Billerica, Massachusetts, USA); nominal molecular weight cutoff: 100 kDa). The purification protocol has previously been described in detail [26].

The conjugates of FPZ with TC-AuNPs (FPZ-AuNPs) were prepared via a ligand exchange reaction following the protocol described for chlorpromazine (CPZ) conjugates with AuNPs [27]. First, a 10 mM aqueous solution of FPZ was prepared. At a constant temperature of 25 °C, an appropriate volume of TC-AuNP suspension, with a controlled nanoparticle mass concentration, was added to the FPZ solution at the desired concentration. This resulted in 10 mL of FPZ-AuNP conjugates with fixed concentrations of FPZ and TC-AuNPs. The TC-AuNPs were added under vigorous stirring, which was maintained for 20 min.

### 3.4. Physicochemical Characteristics of TC-AuNPs and FPZ-AuNP Conjugates

The mass concentration of TC-AuNPs was measured using inductively coupled plasma optical emission spectrometry (ICP-OES) with a Perkin-Elmer OPTIMA 2100DV instrument (Wellseley, MA, USA). For this, 1 mL of the stock suspension was mixed with 5 mL of hot aqua regia to dissolve the TC-AuNPs. The mixture was then diluted with 44 mL of Milli-Q water. The effectiveness of AuNP dissolution was evaluated by analyzing the absorption spectrum of the mixture. The absence of characteristic absorption bands around 530 nm, arising from localized surface plasmon resonance (LSPR) [59], confirmed the complete digestion of TC-AuNPs.

The optical properties and stability of FPZ, TC-AuNPs, and FPZ-AuNP conjugates dispersed in solutions with controlled ionic strength (*I*), pH, and temperature were evaluated based on UV-vis spectra recorded using a UV-2600 spectrometer (Shimadzu, Kyoto, Japan).

The morphology and average size of TC-AuNPs were evaluated by examining micrographs obtained using a JEOL JSM-7500F scanning electron microscope (SEM) (JEOL, Tokyo, Japan) equipped with a transmission electron detector (TED). The images were analyzed with MultiScan v. 18. 3 software, and a histogram was created by measuring the surface area and diameter of 1000 TC-AuNPs.

The hydrodynamic diameter (*d_H_*) and zeta potential (ζ) of TC-AuNPs and CPZ-AuNP conjugates were determined using the Zetasizer Nano ZS (Malvern Instruments, Malvern, UK). The zeta potential was calculated using Henry’s model. Each measurement was conducted under controlled pH, ionic strength, and temperature.

### 3.5. Spectroscopic Characteristics of FZ-AuNP Conjugates

The spectroscopic characteristics included the investigation of spectra recorded with the use of Raman spectroscopy (RS) and surface-enhanced Raman spectroscopy (SERS). RS and SERS measurements of FPZ and FPZ–AuNP conjugates were conducted using an inVia Renishaw spectrometer coupled with a Leica confocal microscope (Renishaw, Wotton-under-Edge, UK) and a 20× magnification objective. A thermoelectrically cooled CCD detector served as the spectral signal detector, while a 632.8 nm laser was used as the excitation source. In the basic experiments, the ionic strength and pH of FPZ–AuNP suspensions were not adjusted by adding additional electrolytes (e.g., sodium chloride). For pH-dependent measurements, hydrochloric acid and sodium hydroxide were used to achieve the desired pH and ionic strength of the FPZ–AuNP suspensions. The suspensions of FPZ-AuNP conjugates were placed on a platinum holder, and the laser was focused on a spot within the drop to collect measurements. The spectra were recorded in three series, with each spectrum (1 scan) being taken over the range of 1800–400 cm^−1^ using 30 s of integration and 100% laser power. The laser’s output power was approximately 2 mW.

### 3.6. Determination of Cytotoxicity of TC-AuNPs and FPZ-AuNP Conjugates Towards SH-SY5Y Cell Line

Cell viability after treatment with FPZ, TC-AuNPs, and FPZ-AuNP conjugates with well-defined compositions was determined using the colorimetric 3-(4,5-dimethylthiazol-2-yl)-2,5-diphenyl tetrazolium bromide assay (MTT assay). SH-SY5Y cells were seeded in 96-well plates at a density of 0.2 × 10^6^ cells per well in a volume of 0.2 mL/well. After 24 h of exposure to FPZ, TC-AuNPs, and the conjugates of a given concentration, 50 μL of an MTT solution (5 mg/L) was added to each well, and they were incubated for 2 h at 37 °C. Subsequently, 0.4 mL of dimethyl sulfoxide (DMSO) was added to the wells. After 5 min, the solutions were centrifuged, and the absorbance of the supernatants at a wavelength of 570 nm was measured using an Epoch microplate reader (BioTek Instruments, Winooski, VT, USA). Cell viability was determined according to a previously described protocol [60].

### 3.7. Theoretical Calculations

The optimization of the FPZ molecule’s geometric structure was conducted based on density functional theory using a local cluster model. Calculations were performed with the Turbomole v7.5 software [61] utilizing the BFGS optimization algorithm [62,63,64,65]. In the calculations, the PBE exchange-correlation gradient potential [66,67] was applied, along with an Ahlirchs def2-QZVPPD basis set for all elements, which, due to its high precision in describing electron distribution in molecules, is essential in calculations of spectroscopic properties [68]. The resulting optimized geometry met the following criteria: (a) the energy difference between successive optimization cycles was below 10^−7^ a.u., (b) the maximum displacement element was under 10^−3^ a.u., and (c) the maximum gradient element was also less than 10^−3^ a.u. Dispersion interactions were included using the Grimme +D3 correction [68]. The resolution of identity (RI) method was employed to calculate electronic Coulomb interactions [69,70]. The COSMO (Conductor-like Screening Model) approximation [71] was used to simulate solvation effects, where the solute molecule is enclosed within a cavity in a dielectric continuum with permittivity ε (ε = ∞ for an ideal solvent) representing the solvent. This cavity was built from the union of spheres with radii R_i_ + R_solv_ for each atom *i*. Vibrational analysis was performed in three stages using the afforce, egrad, and intense programs implemented in the Turbomole 7.5 software.

### 3.8. Data Analysis

For the RS and SERS spectra, a Savitzky–Golay filter (3rd-order polynomial with 5 data points) and a multipoint baseline correction (5 points) were applied. These methods did not affect the relative band intensities. The spectra were normalized using the min–max normalization technique, and the most consistent spectra were selected for further analysis.

The cellular reactions to treatments with FPZ, TC-AuNP, and FPZ-AuNPs were examined across five independent replicates. The obtained data were averaged to calculate the standard deviation. To identify significant differences from the control group, the SAS analysis of variance (ANOVA) procedure was used. Statistical evaluation of the results from each biochemical assay was conducted with the Duncan multirange test, setting the significance level at *p* < 0.05, and it was carried out using PC SAS 8.0 software (SAS Institute, Cary, NC, USA).

## 4. Conclusions

It was found that stable conjugates of FPZ with AuNPs can be produced through electrostatically driven ligand exchange processes. To obtain stable FPZ-AuNP conjugates, the concentrations of citrate-stabilized AuNPs and FPZ were carefully controlled, and the process was carried out under mildly acidic conditions. The electrokinetic properties of FPZ-AuNP conjugates strongly depend on their composition. Moreover, it was demonstrated that the acid–base properties of FPZ are transferred to the FPZ-AuNPs. The isoelectric point of FPZ-AuNPs ranged between 7.0 and 8.5, strongly depending on the amount of FPZ adsorbed onto the AuNP surface.

Insightful spectroscopic characteristics of the Raman spectra (RS) of FPZ and the SERS spectra of FPZ-AuNP conjugates, supported by theoretical calculations, provided valuable knowledge about the conformation of FPZ after adsorption onto the AuNP surface. It was found that FPZ strongly interacts with the gold surface via the piperazine ring and the fluorine atoms of the -CF₃ group substituted on the phenothiazine ring. Based on the SERS spectra, it was also determined that pH changes do not induce the release of FPZ molecules from the AuNP surface.

The adsorption of FPZ molecules on the surface of AuNPs did not result in a decrease in their biological activity. Biological studies showed that FPZ-AuNP conjugates were more toxic to SH-SY5Y cells than negatively charged TC-AuNPs. FPZ-AuNPs containing higher amounts of FPZ were noticeably more toxic to the cells than free FPZ.

## Figures and Tables

**Table 1:** Calculated and experimental wavenumbers together with the bands’ assignments and full width at half maximum (FWHM) for the RS spectrum of FPZ and the SERS spectrum of FPZ-AuNP conjugates [27,34,35,36,37,38,39,40,41,42,43,44,45,46,47,48].

Experimental Wavenumbers				Calculated Wavenumbers	Band Assignment	
RS		SERS				
ν [cm^−1^]	FWHM [cm^−1^]	ν [cm^−1^]	FWHM [cm^−1^]	ν [cm^−1^]	Literature	DFT
		410	12			
435	11	429	19	424	δ_oop_(PTZ), δ_oop_(ϕ), ρ_b_(CNC)_PTZ_	δ_oop_(PTZ), δ_oop_(Pz)
451	10	450	10	456		δ_oop_(Pz), γ_t_(C_10_,C_11_,C_12_)
483	11	479	12	481	ρ_b_(CSC)	δ_oop_(Pz), δ_oop_(PTZ)
527	18	521	14	525		ρ_b_(C_23_H_49_)_PTZ_, ρ_b_(C_24_H_50_)_PTZ_, δ_oop_(Pz)
544	11	541	17	550	δ(CF_3_)	ν(S_20_C_22_)_PTZ_, δ_oop_(PTZ), δ_oop_(Pz)
611	10	604	15	621	δ(CCC)_PTZ_, δ(CNC)_PTZ_	ρ_r_(C_2_H_32_H_33_), ρ_r_(C_3_H_34_H_35_)
		617	12			
		633	11		δ(CF_3_)	
		645	11		ρ_b_(ϕ)	
663	10	656	11	664		δ_oop_(Pz)
679	9	676	13	671	ν_s_(ϕ), ν(CS)	ρ_b_(C_19_H_48_)_PTZ_, ρ_b_(C_18_H_47_)_PTZ_, δ_oop_(Pz)
701	6	699	11	693	ρ_b_(CCC)	ρ_b_(C_25_H_51_)_PTZ_, ρ_b_(C_26_H_52_)_PTZ_, ν(C_11_C_12_)
723	7	725	33	736	δ(ϕ), δ_oop_(CH)_ϕ_	δ_i.p._(Pz)
732	15	749	22	746	ν_s_(CF_2_)	ν_s_(CF_3_)
772	25	770	19		δ_oop_(CH)_ϕ_	δ_i.p._(Pz), ρ_r_(C_2_H_32_H_33_), ρ_r_(C_3_H_34_H_35_)
		800	20			
816	18	818	9	819		δ_i.p._(Pz), ρ_r_(C_3_H_34_H_35_), ρ_w_(C_10_H_40_H_41_),
		837	13			
		854	16			
		871	12			
		887	8			
		903	11			
		920	15			
938	18	936	21	933		δ_i.p._(PTZ)
		962	22			
976	11	977	15		δ_oop_(CH)	ν(C_10_C_11_)
		992	13			
		1018	20			
1039	10	1038	18	1038	δ_ip_(CH)_Ph_, ν_s_(ϕ), ν(CC), ν(CH)	δ_ip_(CH)_PTZ_
1054	16	1053	13	1055		δ_ip_(CH)_PTZ,_ ν(C_12_N_13_)_,_ ν(C_10_C_11_), ρ_b_(CH)_Pz_, ν(O_1_C_2_)
1082	19	1084	10	1080	ν(CSC), ν(NC), ν_s_(CF_3_)	ν(C_17_F_30_)
1110	12	1100	13	1111	ν_as_(CNC), ν_s_(ϕ),	ρ_t_(C_2_H_32_H_33_), ρ_b_(O_1_H_31_), ρ_t_(C_3_H_34_H_35_), ν_s_(Pz)
1131	18	1122	17	1135	δ(CH)_CH2_, δ(CH)_CH3_,	ρ_t_(C_2_H_32_H_33_), ρ_t_(C_3_H_34_H_35_), ρ_b_(CH)_PTZ_
1149	12	1145	7	1141		ν_as_(C_17_F_28_F_28_), ρ_b_(CH)_PTZ_
1163	19	1169	21	1163	ρ_b_(CH)_ϕ_	ρ_b_(CH)_PTZ_, ρ_w_(C_12_H_44_H_45_), ρ_w_(C_11_H_42_H_43_), ρ_w_(C_10_H_40_H_41_), ρ_w_(C_2_H_32_H_33_), ρ_w_(C_3_H_34_H_35_)
		1190	10		ν(CF3)	
		1201	12		ν_s_(CF_2_)	
		1212	13		ν_as_(CNC), ρ_b_(CH)_ϕ_	
		1237	19			
1247	15	1250	12	1242	ν_s_(CNC), ρ_b_(CH)_ϕ_, ρ_t_(CH_2_)	ν(C_16_C_17_), ρ_b_(C_19_H_48_)_PTZ_, ρ_b_(C_18_H_47_)_PTZ_
		1262	9			
1278	11			1277	δ(CH)_CH2_, ρ_r_(CH), ρ_b_(CH)_ϕ_	ρ_w_(C_3_H_34_H_35_), ν(CN)_Pz_
1290	12	1291	24	1300	ρ_b_(CH), ρ_t_(CH_2_), ν(CH), ν(CCC), ρ_b_(CNC)	ρ_b_(CH)_PTZ_, ν(C_16_C_17_)
1305	19	1308	13	1316	ρ_b_(CH), ρ_t_(CH_2_), ν(CH), ν(CCC), ρ_b_(CNC)	ρ_b_(CH)_PTZ_, ν(C_16_C_17_)
1325	21	1323	22		ν(CCC)_Ph_, ν(CNC), δ(ϕ)	
1341	8			1346		ρ_s_(C_3_H_34_H_35_)
		1355	25		δ(CH)_CH2_, δ(CH)_CH3_, ρ_r_(CH)_ϕ_	
		1370	15		ν(ϕ)	
1384	11	1384	17	1389	ν(ϕ)	ρ_b_(CH)_PTZ_
1401	7	1400	19	1392	ρ_w_(CH_2_)	ρ_w_(C_2_H_32_H_33_), ρ_b_(O_1_H_31_), ρ_w_(C_3_H_34_H_35_)
1425	12	1421	22			
		1440	24		δ(CH_3_)	
1454	16	1460	27	1453		ρ_s_(C_2_H_32_H_33_)
1497	11	1492	15			
		1504	13			
		1537	23			
		1562	38			
1574	13			1580	ν(CC)_ϕ_	
1591	8	1587	13		ν(CC)_ϕ_, ν(C=C)	
1603	10	1602	15		ν(CC)_ϕ_, ν(C=C)	
		1620	27			
		1642	24		ν(C=C)	

Abbrevations: PTZ, phenotiazine moiety; Pz, Piperazine moiety; ν, stretching; ρ_b_, bending; ρ_w_, wagging; ρ_s_, scissoring; δ, deformation; s, symmetric; as, antisymmetric; oop, out-of-plane; i.p., in-plane; ϕ, aromatic ring.

## Data Availability

The data presented in this study are available in the Supplementary Materials.

## Supplementary Materials

The following supporting information can be downloaded at https://www.mdpi.com/article/10.3390/molecules29245948/s1, Figure S1: Absorption spectra of TC-AuNP suspensions; Figure S2: The dependence of the hydrodynamic diameter of TC-AuNPs on (a) ionic strength and (b) pH; Figure S3: The dependence of the zeta potential of TC-AuNPs on pH; Figure S4: Absorption spectra of aqueous solutions of FPZ; Figure S5: Absorption spectra of aqueous solutions of FPZ recorded for diverse pH levels; Figure S6: Absorption spectra of aqueous solutions of FPZ-AuNP conjugates.
