# Supplementary material for: Spectroscopic Properties and Biological Activity of Fluphenazine Conjugates with Gold Nanoparticles
Source: Molecules. 2024 Dec 17;29(24):5948. doi: 10.3390/molecules29245948 (PMC11676885; doi:10.3390/molecules29245948)
Supplement: Supplementary file 1 [file molecules-29-05948-s001.zip › molecules-3346347-supplementary.pdf]

Supporting materials

Spectroscopic properties and biological activity of fluphenazine conjugates with gold nanoparticles

Oliwia Kowalska[1] (oliwia.kowalska@ikifp.edu.pl), Natalia Piergies[2] (natalia.piergies@ifj.edu.pl), Anna Barbasz[3] (anna.barbasz@uken.krakow.pl), Piotr Niemiec[4] (p_niemiec@anstar.edu.pl), Patrycja Gnacek[1] (patrycja.gnacek@ikifp.edu.pl), Dorota Duraczyńska[1] (dorota.duraczyńska@ikifp.edu.pl), Magdalena Oćwieja[1*] (magdalena.ocwieja@ikifp.edu.pl)

[1]*Jerzy Haber Institute of Catalysis and Surface Chemistry, Polish Academy of Sciences, Niezapominajek 8, PL-30239 Krakow, Poland*

[2]*Institute of Nuclear Physics Polish Academy of Sciences, PL-31342 Krakow, Poland*

[3]*Department of Biochemistry and Biophysics, Institute of Biology and Earth Sciences, University of the National Education Commission, Podchorazych 2, PL-30084, Krakow, Poland*

[4]*Faculty of Mathematics and Natural Sciences, Department of Chemistry, University of Applied Sciences in Tarnow, Mickiewicza 8, 33-100 Tarnow, Poland*

* Corresponding author
Magdalena Oćwieja
Jerzy Haber Institute of Catalysis and Surface Chemistry, Polish Academy of Sciences
Niezapominajek 8, PL-30239 Krakow, Poland
email: magdalena.ocwieja@ikifp.edu.pl
phone: +48126395112

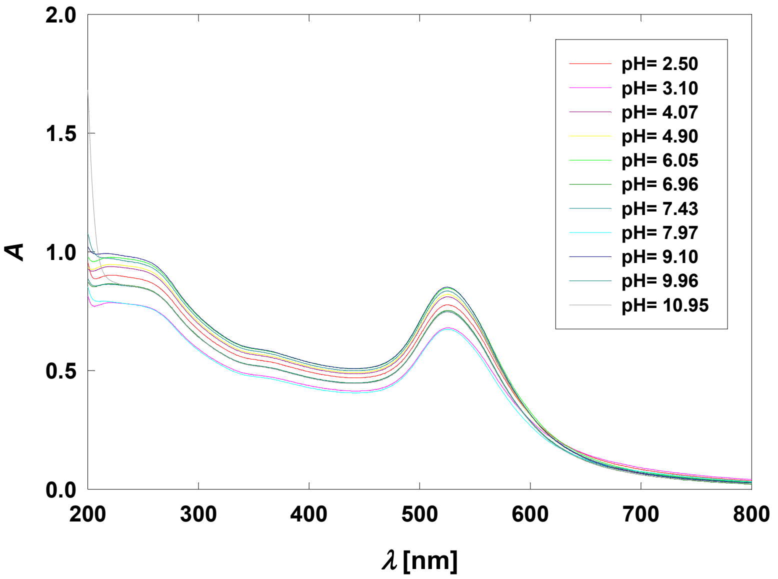

Fig. S1. Absorption spectra of TC-AuNP suspensions (40-50 mg/L) recorded at various pH values, adjusted by the addition of HCl or NaOH.

a)

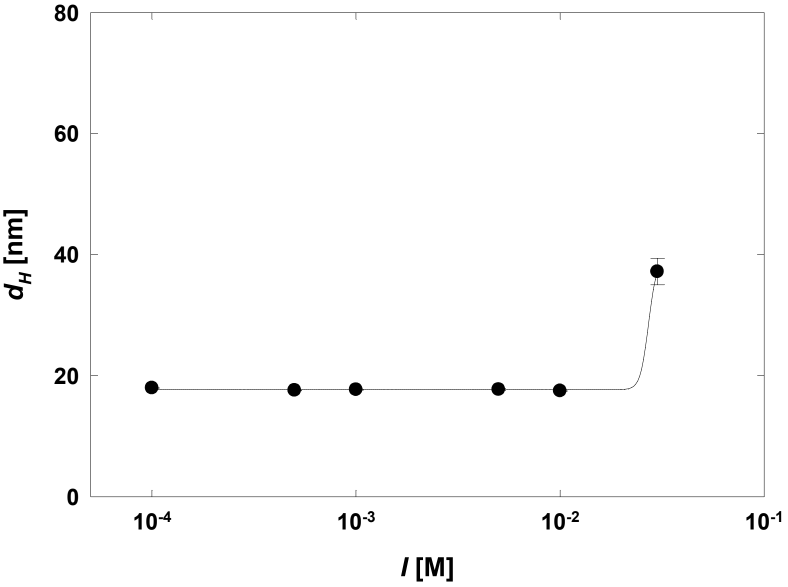

b)

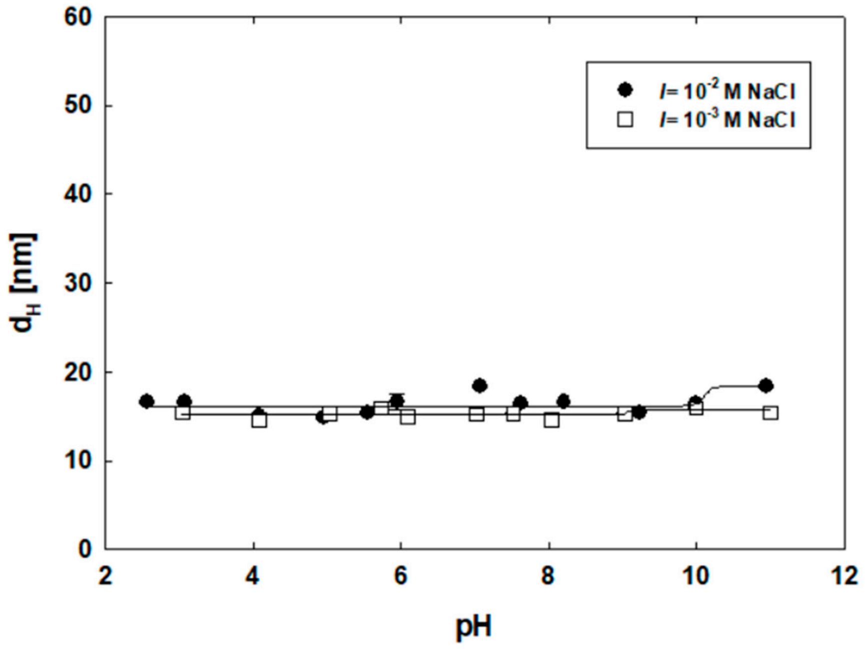

Fig. S2. The dependence of the hydrodynamic diameter of TC-AuNPs on **a)** ionic strength, measured at pH = 5.8, and **b)** pH, measured at ionic strength of 10^{-2} M and 10^{-3} M NaCl. The measurements were conducted at a temperature of 25° C using a TC-AuNP suspension with a concentration of 50 mg/L. The solid lines serve as a guide for the eye.

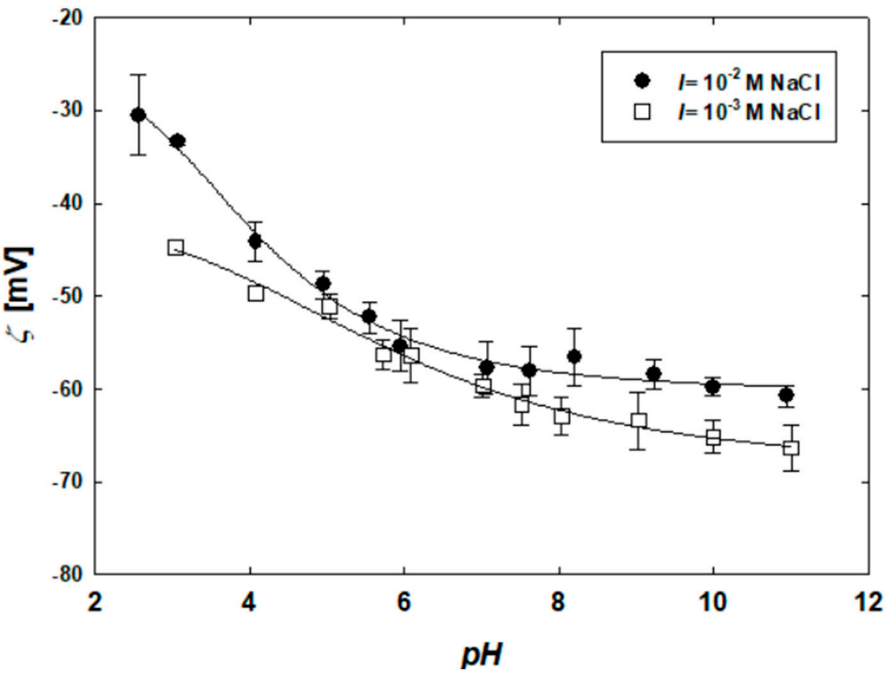

Fig. S3. The dependence of the zeta potential of TC-AuNPs on pH, determined at ionic strengths of 10^{-2} and 10^{-3} M. The measurements were conducted at temperature of 25° C using TC-AuNP suspension with a concentration of 50 mg/L. The solid lines serve as a guide for the eyes.

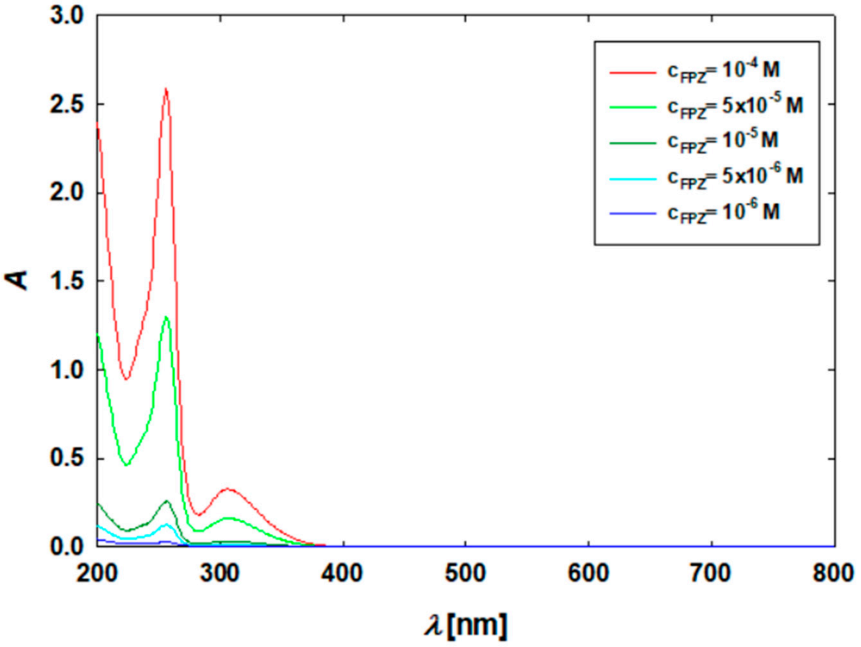

Fig. S4. Absorption spectra of aqueous solutions of FPZ (in the form of fluphenazine hydrochloride) recorded for various FPZ concentrations.

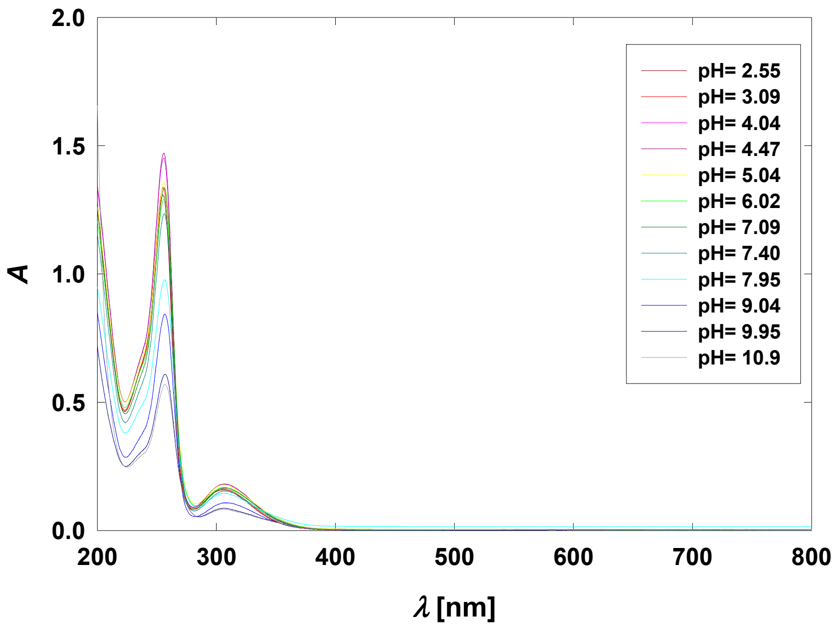

Fig. S5. Absorption spectra of aqueous solutions of FPZ (in the form of fluphenazine hydrochloride) recorded for various pH values, adjusted by the addition of HCl or NaOH. The concentration of FPZ in the solutions was 5×10^{-5} M.

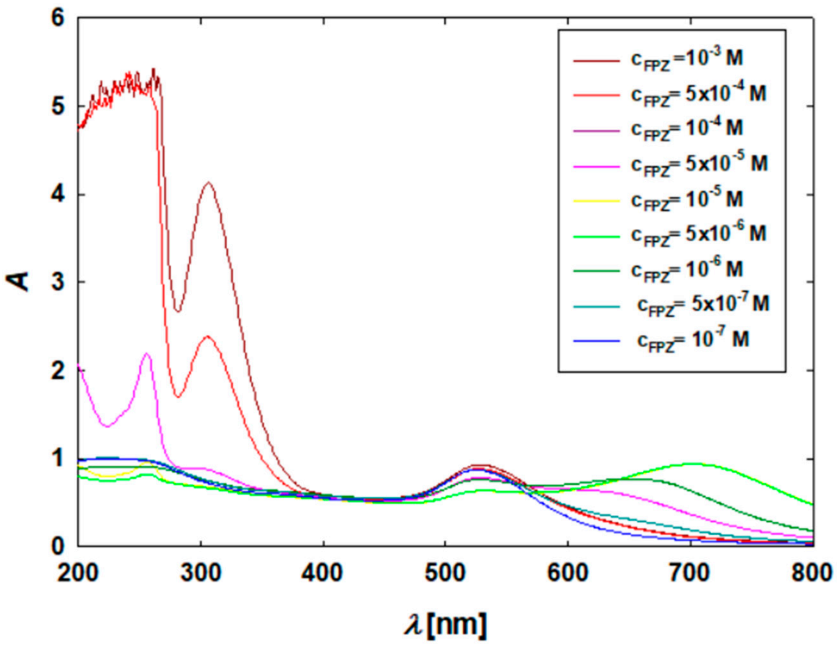

Fig. S6. Absorption spectra of aqueous solutions of FPZ-AuNP conjugates with diverse compositions recorded using a TC-AuNP concentration of 50 mg/L in each experiment.
